# Indoor Carbon Monoxide: A Case Study in England for Detection and Interventions to Reduce Population Exposure

**DOI:** 10.1155/2013/735952

**Published:** 2013-04-04

**Authors:** L. J. McCann, R. Close, L. Staines, M. Weaver, G. Cutter, G. S. Leonardi

**Affiliations:** ^1^Centre for Radiation, Chemical and Environmental Hazards, Health Protection Agency, Chilton, Didcot, Oxon OX11 0RQ, UK; ^2^Field Epidemiology Training Programme, Health Protection Agency, UK; ^3^European Programme for Intervention Epidemiology Training (EPIET), European Centre for Disease Prevention and Control (ECDC), Stockholm, Sweden; ^4^Hackney Homes, London E8 1BJ, UK; ^5^London School of Hygiene and Tropical Medicine, London WC1E 7HT, UK

## Abstract

*Background*. Potential exposure to carbon monoxide (CO) in private homes is largely unquantified. *Aim*. To estimate prevalence of potential exposure to CO in residential dwellings and describe associated interventions in an inner-city community. *Methods*. A housing association in London, Hackney Homes, began fitting CO alarms in the 22,831 local authority homes it is responsible for in January 2010. A gas engineer investigated each alarm activation and recorded the information on a standard form. We undertook a cross-sectional study of all 22,831 homes, using data from these forms. Descriptive analysis was performed, including incidence, monthly variation, cause of alarm activation, and actions taken. *Results*. Between November 2011 and April 2012, 106 incidents were reported. Of these, 34.6% identified an issue with a gas appliance, and 10.6% identified misuse of cooking methods as the cause of activation. Relevant interventions were put in place, including disconnection of the gas appliance and education around cooking methods. *Discussion*. Little is known about the burden of CO poisoning in residential dwellings. This study provides important information on the path to quantifying population exposure to CO as well as establishing a possible approach to access this key information and realistic interventions to reduce potential exposure.

## 1. Introduction

Carbon monoxide (CO) is a potentially fatal, colourless, odourless, and tasteless gas that results from the incomplete combustion of carbon-containing fuels. In England and Wales, an estimated 40 people (range 25–45 between 2006 and 2011) die [1], and over 200 are admitted to hospital each year from accidental CO poisoning; a further 4000 present to Accident and Emergency Departments but are not admitted [2]. However, these figures are likely to be underestimated as many more people are thought to be exposed to CO and suffer from CO poisoning but remain undiagnosed or even misdiagnosed due to its nonspecific symptoms [3] which include headache, tiredness, and nausea. More severely, CO poisoning can lead to cognitive impairment, convulsions, unconsciousness, and even death. CO poisoning therefore represents a significant public health issue [4, 5]. 

There is an increasing recognition that the burden of morbidity and mortality relating to accidental CO poisoning may be far greater than that documented, particularly as there is little evidence to quantify those who do not even present to a healthcare establishment. However, the extent of this burden and the success of intervention methods are still unknown. A report by the All-Party Parliamentary Gas Safety Group (now the All-Party Parliamentary Carbon Monoxide Group) [6] emphasised the urgent need for research into the effects and prevalence of CO, in order to improve identification of the presence of CO and to provide a more accurate picture of the true number of people affected. 

In order to estimate the burden of disease for CO, understanding potential hazards for exposure is as important as quantifying those who become ill due to CO poisoning. Hazards such as exposure from using a generator indoors are well established [7]; however, the burden of disease in the community (such as private dwellings) is less well evidenced. Karalliedde and Keshishian [8] report that the most common source of CO poisoning in the home is faulty heating or cooking appliances, and therefore CO exposure in this setting is almost entirely preventable, particularly if CO detectors are installed [9]. There are currently no reliable prevalence data to indicate the extent of the problem in the community [8]. Croxford et al. [10] looked into the association between the risk of CO exposure at low concentration and health outcomes. They found that unsafe gas appliance installations were linked to an increased risk of suffering with at least one self-reported neurological symptom, supporting the need for further research into community exposure to assess the level of burden. Another study [11] carried out continuous monitoring of levels of CO exposure in homes and found that nearly one-fifth of lower-income families could be regularly exposed to level of CO which exceeds the World Health Organisation (WHO) guidelines. The WHO guideline values (ppm values rounded) and periods of time-weighted average exposures have been determined in such a way that the carboxyhaemoglobin level of 2.5% is not exceeded, even when a normal subject engages in light or moderate exercise: 100 mg/m^3^ (87 ppm) for 15 min; 60 mg/m^3^ (52 ppm) for 30 min; 30 mg/m^3^ (26 ppm) for 1 hour; 10 mg/m^3^ (9 ppm) for 8 hours [12].

The purpose of this pilot study was to investigate the incidence of and reasons for CO events recorded when CO alarms are triggered and the interventions put in place.

## 2. Methods

### 2.1. Setting and Study Population

Hackney is an inner city borough of London, situated in the north east of the capital with a population of 219,200 [13], and is the second most deprived local authority in England [14]. In 2010, there were 98,950 dwellings in Hackney; of which 23% are owned by the Hackney Council [15]. From 2006, all the local authority owned housing within Hackney (*n* = 22, 831) was managed by Hackney Homes, an arm's length management organisation. In 2010, Hackney Homes decided that it would establish a programme to monitor CO in the homes under its care, as part of its partnership and contractual arrangements established with the local borough council of Hackney for the management of social housing in that area of London. Trained gas engineers employed by Hackney Homes used an agreed protocol to place at least one CO alarm in all homes they were responsible for starting from January 1st, 2010.

### 2.2. Study Design

A cross-sectional study was used for this pilot to inform possible hypotheses to be evaluated in further analytical studies [16]. The main measurement in this study concerned the hazard to health (i.e., a CO alarm being set off).

### 2.3. Detection of CO in the Community

The study population under investigation was all local authority homes managed by Hackney Homes which have had a CO alarm fitted (*n* = 22, 831). The CO alarms (Fire Angel Model CO-9X) contain an electrochemical cell designed to detect a concentration of CO above 50 ppm in ambient air for 60 to 90 minutes, above 100 ppm for 10 to 40 minutes, and above 330 ppm within three minutes following the initiation of exposure, as stipulated by the British standard BS EN 50291 [17, 18]. The CO alarms are powered by a nonremovable integrated lithium power pack to prevent the occupants tampering with the battery and are expected to last 7 years in normal operating conditions. The device sounds a 85 dB alarm at 1 metre and flashes LED light to alert occupants in case of CO detection. Alarms were installed in every room containing a fuel burning appliance, with a maximum of three alarms fitted per household, and they were placed between 1 m and 3 m from the potential source (e.g., boiler, gas cooker, and gas fire) and fixed to the wall. 

Following activation of an alarm, the occupant informs National Grid and Hackney Homes. National Grid sends a staff member to the property to make it safe (e.g., turn off the gas supply). Within two days, Hackney Homes sends a qualified gas engineer to the property to investigate the alarm activation. Gas engineers employed for this role are required to have obtained registration with a professional body (Gas Safe). At each visit, the gas engineer conducted CO tests on each gas appliance present in the dwelling as well as outside the property and collected information on the occupants' reasons for concern, including whether anyone was suffering illness, any faults identified, interventions undertaken, and general observations (such as housing type). The information collected is then recorded on a *Gas Safe Fumes Investigation Report*, which is designed to support the standard collection of information from each alarm activation. If an appliance is found to be defective, it is always isolated regardless of the appliance. All incidents reported to Hackney Homes are investigated. 

### 2.4. Data Collection and Study Period

All incidents investigated by Hackney Homes between November 2011 and April 2012 were included in the study. November 2011 was selected as the start of the study period as this was the date when Hackney Homes estimated that all its homes had been fitted with at least one CO alarm and therefore the study population would have remained constant during the study period. Occupant data included in *fumes investigation reports* for the study period were anonymised by Hackney Homes before being sent to the Health Protection Agency where they were entered onto a Microsoft Access 2007 Database. 

### 2.5. Statistical Analysis

Incidents where a gas engineer was called out but did not enter the property and therefore did not carry out an assessment were excluded from further analysis. Photocopied forms where none of the information could be read were also excluded. Photocopied forms where only some of the information could not be read were included, providing the date of inspection that could be read, but the unreadable fields were coded as missing. Data entered onto the database were verified, checked, and cleaned. Data were coded to identify what the cause of the alarm was and what actions were taken or advice was issued. Descriptive analysis was undertaken to calculate incidence, monthly variation, proportion of incidents where the householder reported illness, cause of alarm activation, and actions taken.

## 3. Results

Between November 2011 and April 2012, 106 incidents were reported to Hackney Homes where the CO alarm was activated. The incidence rate was 4.64 incidents per 1000 households for this six-month period. Two incidents were excluded from further analysis as the gas inspector did not gain access to the home and therefore no test was carried out. During the six-month period investigated, November reported the highest number of incidents (31), and the remaining months (December 2011–April 2012) reported between 13 and 17 incidents each month. In 6.7% of incidents, the occupant answered yes to the question “Was anyone suffering from illness?”

There were several issues highlighted during investigation of the incidents as being possible reasons for the alarm being activated (Table 1). More than one issue was identified in 11 incidents (10.6%). One of the main causes of CO alarm activation was that a gas appliance in the home was found to be unsafe due to emitting CO above guideline values when tested by gas engineers. Of the 104 incidents investigated, 36 (34.6%) found that at least one gas appliance (boiler, cooker, or fire) was defective and disconnected. Not all homes had a boiler, a gas cooker, and a gas fire, and therefore the denominator for each gas appliance differed. Of the 104 incidents investigated, 97 noted that a gas cooker was present in the property, and 102 noted that a boiler was present and four noted a gas fire was present. Taking this into account, 9.8% of all incidents had the boiler disconnected, 29.9% had the cooker disconnected, and 25% had the gas fire disconnected. It is important to note, however, that there were four incidents where both the boiler and the cooker were found to be unsafe and therefore disconnected. In 28 incidents (26.9%), the alarm was noted as being faulty (including low on battery) and/or replaced with a new CO alarm. In 12 incidents (11.5%), the CO alarm was reported to be fitted in an unsuitable place (e.g., directly above the cooker). Some of these may have been installed before the programme to fit all homes with an alarm had started. Of these 12 incidents, ten had an additional issue identified on the form, and therefore the positioning of the alarm was likely to be a secondary issue. Eight of these ten incidents also had a gas appliance disconnected by the visiting engineer and two identified misuse of cooking methods as a possible issue. Overall, one-tenth of incidents (10.6%) identified misuse of cooking as the cause of the CO alarm going off. This included using large pans on the cooking hob rings, using foil around the cooking hob ring, using charcoal on the hot plate, and bringing a barbeque indoors.

The gas inspectors also identified further issues which were cause for concern but not likely to be causing CO leakage or to be the cause of the CO alarm activation. Examples include issues with the boiler, such as a new part required or the boiler being scaled up, no flame supervision device (FSD) or stability device on the cooker, and the gas supply to the appliances being undersized. The main interventions put in place are listed in Table 2.

## 4. Discussion

Little is known about the burden of CO poisoning in private dwellings; this study has documented aspects relevant to this at a specific location by using data from CO alarm incidents in local authority homes in the borough of Hackney. Between November 2011 and April 2012, 4.64 incidents per 1000 households were reported (*n* = 106). One of the main findings from this study is that in over one-third of incidents, an issue was identified with at least one gas appliance. This finding has important implications given that Croxford et al. [10] showed that the presence of an unsafe gas appliance installation was linked to increased risk of suffering neurological symptoms. Furthermore, although boilers are considered one of the commonest hazards for CO exposure at home, this study has highlighted that cookers are also a major source of CO exposure at home, both in terms of faulty cookers giving off excess CO and methods of cooking which produce excessive amounts of CO. Interventions undertaken by gas inspectors included disconnection of gas appliances and education concerning cooking methods. It is important to note that some boilers were isolated due to poor flue performance results but were not found to be leaking CO. Some were isolated along with gas cookers and this has no bearing on the inspection of the appliance by Hackney Homes, as defects may have occurred some time after the inspection was carried out.

A number of incidents identified an issue with the CO alarm. Twelve incidents (11.5%) reported that the alarm was not placed in a location according to the protocol. Although ten of these 12 incidents identified an additional issue which was more likely to be the reason for alarm activation (gas appliance unsafe and cooking misuse), it is important to recognise that CO alarms must be fitted in the correct place and by reputable contractors to ensure effective use. The finding that illness in the household was reported for 6.7% of incidents is likely to be due to seasonal background levels, and therefore no link can be made between potential exposure and health effects in this study.

There were a number of limitations with this study which need to be considered. First, we could not document the relationship between detection of CO concentrations and the WHO guideline values [12], which are established to protect the population not only from exposures of short duration, but also from lower exposure of longer duration that are nevertheless recognised to be toxic. The design of the devices included in this study is intended to trigger an alarm only when 50 ppm for 60–90 minutes is reached, 100 ppm for 10–40 minutes, or when dangerously high levels of 300 ppm or more where the alarm will sound within 3 minutes. Therefore, all exposures at a concentration lower than 50 ppm but over a longer time period, which may be causing chronic CO poisoning, were missed by the survey. We may also have missed alarm activations where the occupant did not report the incident to Hackney Homes or National Grid and so the incident was not investigated. If the occupant ventilates the dwelling causing CO to disperse, the alarm will reset itself. We are unable to specify how often this occurs though suspect the number to be low due to the fact that occupants are likely to be concerned about what caused the alarm activation and would want it to be investigated.

Second, the relationship between concentrations of CO in indoor air and personal exposure could not be directly estimated in our study. The Environmental Health Criteria 213 on CO suggests that individual personal exposure does not directly correlate with CO concentrations determined by using fixed site monitors alone [12]. Therefore, although our study has shown that on certain occasions CO concentration exceeded WHO guidelines resulting in an alarm being activated, it does not necessarily mean that CO carboxyhaemoglobin concentration in occupants was elevated.

 A further limitation of the study concerns the representativeness of the study population and validity of any extrapolation of the results to the whole of the UK population. However, the results do provide evidence of the potential exposure to CO which exists in private dwellings and associated interventions to reduce it, particularly in local authority housing.

Despite these limitations, this study has provided important information on the path to quantifying CO exposure in this population. Little is known about the burden of CO particularly in private homes, due in part to a lack of systematic surveillance in the UK and many other countries. Often the more severe acute poisonings are known about and reported to healthcare establishments, but the less severe, more chronic poisonings are less well documented. This study has also established a possible approach to access key information on population exposure to CO and realistic interventions to reduce it. This has important implications for future studies investigating potential CO exposure in private homes aimed at quantifying population exposure to CO, as well as for CO policymakers and those developing CO awareness campaigns or targeted interventions. In particular, this information might be relevant to public health practitioners who design, develop, or implement systems for public health surveillance and tracking of CO-related hazards to health [19, 20]. Given that CO poisoning is difficult to diagnose and can often be missed by doctors due to the patients presenting with symptoms which mimic other common diseases (e.g., flu-like illness and cognitive impairment), understanding potential exposure and possible hazards is particularly important. This work concentrated on one aspect of CO exposure, providing improved understanding of potential community exposure and of environmental hazards for CO, at home. Combining this work with other relevant studies, looking at mortality, hospital admissions, accident and emergency presentations, and GP consultations, will provide a more accurate picture of the burden of disease of CO and could contribute to establishing routine surveillance of CO. The study highlights the need to develop further research in this area in order to develop understanding of the mechanisms involved in CO poisoning in private homes and the burden of disease in these settings. It is recommended that this study population should be followed up over a longer time period. Future studies on CO poisoning in the community should consider sampling a representative population and collect data on socioeconomic status of occupants, housing tenure, characteristics of the homes, and comparisons between urban and rural environments. Further work is also needed to investigate the association between exposure and health. Work in this area will contribute to developing relevant public health actions.

## Figures and Tables

**Table 1:** Issues/faults identified during investigation of CO alarm incidents, November 2011 to April 2012 (*n* = 104).

Issue identified	*N*	%*
(1) Cooker defective	29	29.9
(2) Boiler defective	10	9.8
(3) Fire defective	1	25
(4) Misuse of cooker/cooking methods	11	10.6
(5) CO alarm faulty/low battery/wrongly sited	40	38.5

*Denominator for issues 1, 2, and 3 changed depending on whether each gas appliance was reported as present and tested. Number of incidents where a gas cooker was reported as present and tested: 97; number of incidents where a gas boiler was reported as present and tested: 102; number of incidents where a gas fire was reported as present and tested: 4.

**Table 2:** Main interventions put in place following investigation by gas engineer.

Disconnection of the gas appliance (cooker, boiler, or fire)	
Replacing CO alarm with new alarm	
Resiting of current CO alarm	
Providing advice to tenant on size of pans when cooking	
Providing advice to tenant on other misuse of cooker (e.g. placing foil around the gas hob)	
Providing advice to tenant on other CO producing activities (e.g. bringing a barbeque inside the house or smoking a shisha pipe)	
Providing advice to tenant on ventilating the kitchen when cooking	
Reiterating that it is mandatory for all cookers in flats to have flame supervision devices	
